# *statgenMPP*: an R package implementing an IBD-based mixed model approach for QTL mapping in a wide range of multi-parent populations

**DOI:** 10.1093/bioinformatics/btac662

**Published:** 2022-10-04

**Authors:** Wenhao Li, Martin P Boer, Bart-Jan van Rossum, Chaozhi Zheng, Ronny V L Joosen, Fred A van Eeuwijk

**Affiliations:** Biometris, Wageningen University and Research Center, Wageningen, 6700 AC, The Netherlands; Biometris, Wageningen University and Research Center, Wageningen, 6700 AC, The Netherlands; Biometris, Wageningen University and Research Center, Wageningen, 6700 AC, The Netherlands; Biometris, Wageningen University and Research Center, Wageningen, 6700 AC, The Netherlands; Rijk Zwaan Breeding B.V., De Lier 2678 ZG, The Netherlands; Biometris, Wageningen University and Research Center, Wageningen, 6700 AC, The Netherlands

## Abstract

**Motivation:**

Multi-parent populations (MPPs) are popular for QTL mapping because they combine wide genetic diversity in parents with easy control of population structure, but a limited number of software tools for QTL mapping are specifically developed for general MPP designs.

**Results:**

We developed an R package called *statgenMPP*, adopting a unified identity-by-descent (IBD)-based mixed model approach for QTL analysis in MPPs. The package offers easy-to-use functionalities of IBD calculations, mixed model solutions and visualizations for QTL mapping in a wide range of MPP designs, including diallele, nested-association mapping populations, multi-parent advanced genetic inter-cross populations and other complicated MPPs with known crossing schemes.

**Availability and implementation:**

The R package *statgenMPP* is open-source and freely available on CRAN at https://CRAN.R-project.org/package=statgenMPP

**Supplementary information:**

Supplementary data are available at *Bioinformatics* online.

## 1 Introduction

Multi-parent population (MPP) designs capture wide genetic diversity and overcome the drawbacks of low minor-allele frequencies and population structures (Arrones *et al.*, 2020; Scott *et al.*, 2020). General MPPs, e.g. diallele (Coles *et al.*, 2010; Giraud *et al.*, 2017), NAM (Yu *et al.*, 2008) and MAGIC (Gardner *et al.*, 2016; Huang *et al.*, 2015) are nowadays widely used in genetic studies and plant breeding programs. Statistical models that can be used for MPP designs are either family-based (linkage mapping) or population-based (linkage disequilibrium) methods (Giraud *et al.*, 2014; Würschum *et al.*, 2012; Xu *et al.*, 2017), but a limited number of software tools is specifically developed for MPP designs.

In this Application Note, we present an easy-to-use R package, *statgenMPP*, adopting an identity-by-descent (IBD)-based mixed model approach for QTL mapping. Compared to other tools, *statgenMPP* integrates a framework of IBD calculation (Boer and van Rossum, 2021b; Zheng *et al.*, 2014, 2015) with linear mixed models (Boer and van Rossum, 2021a). The IBD-based mixed model approach estimates random QTL effects in relation to IBD probabilities of parental origins across the offspring genome (Li *et al.*, 2021; Wei and Xu, 2016) while accounting for polygenic and family background genetic variation, which has been proven to increase the mapping power and resolution of QTLs for simulated and empirical MPPs (Li *et al.*, 2021). Single crosses can also be analyzed with *statgenMPP*, for a full list of available population types, see Boer and van Rossum (2021b).

## 2 Materials and methods

**Fig. 1.**
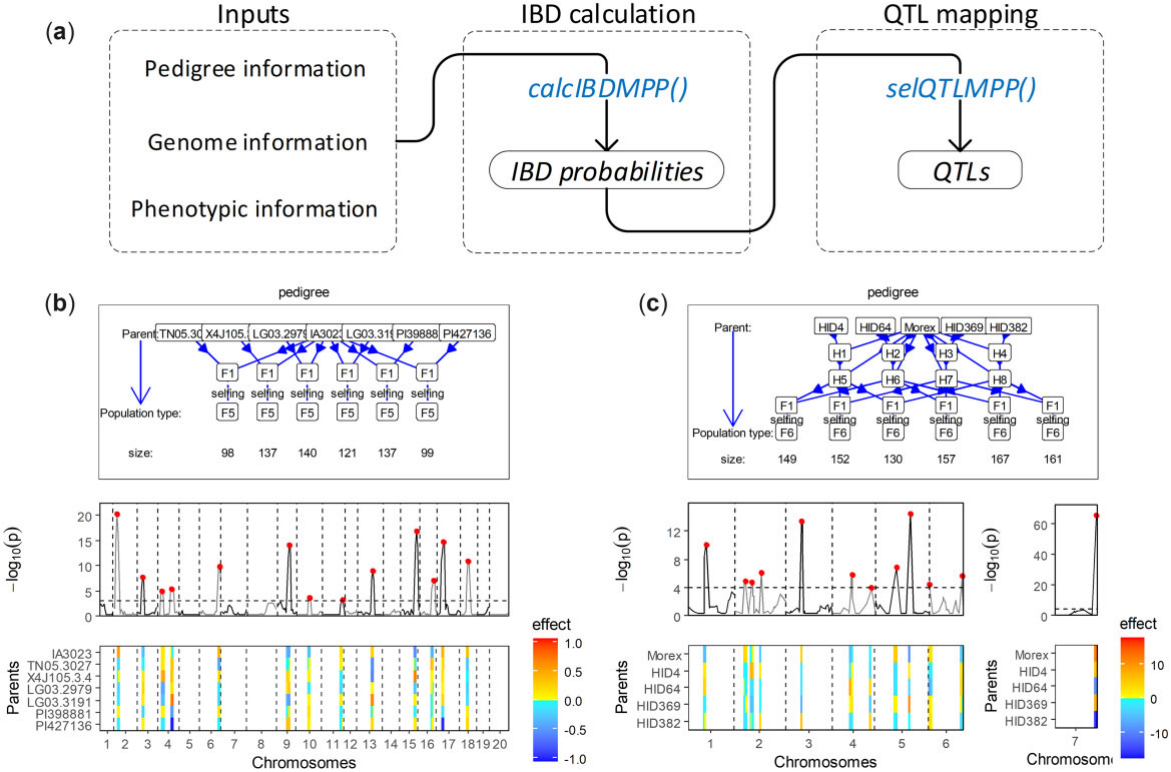
(**a**) The workflow of IBD-based QTL mapping implemented in statgenMPP. QTL mapping in the examples of (**b**) soybean NAM design for seed weight and (**c**) barley complex design for awn length. Upper panel pedigree plots of MPP designs. Middle panel QTL mapping profiles expressed at −log10(p) scale. Lower panel parental QTL effects

Figure 1a demonstrates the workflow of IBD-based QTL mapping using *statgenMPP* that comprises two main steps: IBD calculation and QTL mapping.

### 2.1 IBD calculation

The framework of hidden Markov models (HMM) and inheritance vectors (Zheng *et al.*, 2014, 2015; Boer and van Rossum, 2021b) is employed for the IBD calculation. For a wide range of MPP designs, IBD probabilities are calculated by the function *calcIBDMPP()* at a customizable grid (cM) for the specified population type. For complex MAGIC designs and designs with complicated pedigree structures, the IBD probabilities can be first calculated using RABBIT (Zheng, 2019) and then imported by the function *readRABBITMPP()*.

### 2.2 QTL mapping

IBD probabilities between parents and offspring, as design vectors, or genetic predictors, are fitted in a mixed model for QTL mapping using the function *selQTLMPP()*. The IBD-based mixed model approach is described by Li *et al.* (2021). To test each position on a 1D grid along the genome, a single locus QTL model is fitted whose effects are modeled as random in a mixed effects model:

Y=Xβ+Mqaq_+g+ε_aq_∼MVN(0,IPσq2), g_∼MVN(0,Kσg2),ε¯∼MVN(0, ⊕k=1FInkσεk2)

Y is a vector with phenotypes; X is the design matrix indicating to which family each individual belongs; β is a vector of fixed family intercepts; $M_{q}$ is the design matrix containing the expected number of parental alleles as a function of IBD probabilities; $a_{q}$ is the vector of random parental effects with the variance–covariance (VCOV) structure $I_{P}\;\sigma_{q}^{2}$, in which $I_{P}\;$is the identity matrix for P parents and $\sigma_{q}^{2}$ is the genetic variance of the QTL effects; it is optional to include a polygenic term g whose VCOV is described by the kinship matrix K; the residual term ε has a family-specific VCOV structure ${⊕}_{k=1}^{F}I_{n_{k}}\sigma_{\varepsilon_{k}}^{2}$ in which $\sigma_{\varepsilon_{k}}^{2}$ is the residual variance of individuals in the *k*th family (k=1,2,...,F)with family size $n_{k}$.

The linear mixed model is fitted and variance components are estimated based on restricted maximum likelihood. Variance components corresponding to putative QTLs ($\sigma_{q}^{2}=0$ versus $\sigma_{q}^{2}\neq 0$) are evaluated by likelihood ratio tests (LRT) that approximate a mixture of $\chi^{2}$ distributions (Self and Liang, 1987). Multiple rounds of genome QTL scans can be performed until either (i) no new QTL outside of a certain window size is found with a −log10(p) value below a predefined threshold or (ii) a predefined maximum number of QTLs is reached.

## 3 Applications

We demonstrate the main functionalities of *statgenMPP* using two publicly available MPP designs—a soybean NAM design and a barley complex design (datasets are provided in the supplementary material with R codes). Other examples with full details are available in the vignette of *statgenMPP*.

All computations were performed in (64-bit) R 4.2.1 (R Core Team, 2022) and a 3.10 GHz Intel Core i5 processor computer with 16GB of RAM and Windows 10 operating system. We used the parallel option of *statgenMPP* using four cores, and using the default values, not including a kinship matrix.

We selected six families (Fig. 1b, upper panel) with a total population size of 732 genotypes from the soybean NAM project (https://soybase.org/SoybeanNAM/index.php) (Xavier *et al.*, 2018) for analysis to demonstrate the functionalities of *statgenMPP*. The consensus map containing 4289 markers (Song *et al.*, 2017) was used to calculate IBD probabilities on a regular 5 cM grid of evaluation points. We map QTLs for the trait of seed weight (‘mean_seedWT’) as an example. The total computation time was 1.03 min. The second example is a complex barley MPP design (Liller *et al.*, 2017), with a total population size of 916 genotypes, for QTL mapping for awn length (‘Awn_length’) (Fig. 1c, upper panel). The total computation time was 1.43 min. IBD calculation for the complex design was performed by RABBIT, and then the output was imported by the *readRABBITMPP()* function in *statgenMPP*.

Results of QTL mapping for soybean NAM and barley complex MPP designs are shown in Figure 1b and c. For example in the barley complex design, all QTLs for awn length in the barley complex design can be confirmed from the previous study where the strong QTL on chromosome 7 was successfully fine mapped (Liller *et al.*, 2017). Further details on how to use the package for visualization can be found in the *statgenMPP* vignette.

## 4 Conclusion

An increasing range and number of MPP designs become available for QTL mapping in breeding programs and genetic studies. We introduce the R package *statgenMPP* that covers the demands for versatile QTL analysis in MPP designs. *statgenMPP* contains a unified IBD-based mixed model framework for mapping multi-allelic QTLs. We analyzed multiple MPP data sets with *statgenMPP*, whose data are available within the package, and demonstrated the theoretical and practical advantages of our approach. Extensions of *statgenMPP* will deal with epistasis, pleiotropy and QTL-by-environment analysis, to allow the user to explore the full potential of MPP designs.

## Supplementary Material

btac662_Supplementary_Data

## Data Availability

All data are incorporated into the article and its online supplementary material.
